# Stromal Vascular Fraction Gel for Osteoarthritis: Mechanisms and Therapeutic Benefits

**DOI:** 10.1155/sci/8316087

**Published:** 2026-08-28

**Authors:** Runze Zhang, Yuanhang Yang, Wanli Gao, Xing Lei, Xin Yin, Wei Zhang, Ling Zhang, Yunqi Zhang, Maohua Chen, Junjun Yang, Lisheng Wu

**Affiliations:** ^1^ Orthopedic Medical Center, Linyi People’s Hospital, Shandong Second Medical University, Linyi, Shandong, China, ly120.cn; ^2^ School of Mechanical and Aerospace Engineering, Nanyang Technological University, Singapore 639798, Singapore, ntu.edu.sg; ^3^ College of Artificial Intelligence Medicine, Chongqing Medical University, Chongqing, China, cqmu.edu.cn; ^4^ Department of Pharmacy, The Second Affiliated Hospital, Army Medical University (Third Military Medical University), Chongqing, China, tmmu.edu.cn; ^5^ Key Laboratory of Biorheological Science and Technology, Ministry of Education College of Bioengineering, Chongqing University, Chongqing, China, cqu.edu.cn; ^6^ Center for Joint Surgery, Southwest Hospital, Army Medical University (Third Military Medical University), Chongqing, China, tmmu.edu.cn

**Keywords:** osteoarthritis, precision therapy, regenerative medicine, stromal vascular fraction gel

## Abstract

Osteoarthritis (OA), a prevalent degenerative joint disorder, is suboptimally managed by existing treatments owing to adverse effects, limited therapeutic efficacy, and the inability to achieve genuine cartilage repair. Adipose‐derived stromal vascular fraction (SVF)‐gel, which is abundant in stem cells and bioactive factors, has an autologous origin and favorable immunocompatibility and exhibits favorable safety and efficacy in facilitating cartilage regeneration and repair. Building upon prior research, this review discusses the clinical efficacy and potential of SVF‐gel, while also recognizing challenges such as the standardization of preparation procedures and dosage optimization. Future research ought to concentrate on integrating advanced technologies, including gene editing, artificial intelligence (AI), and nanomaterials, to drive its development towards more precise and personalized therapeutic approaches.

## 1. Introduction

**Figure 1:**
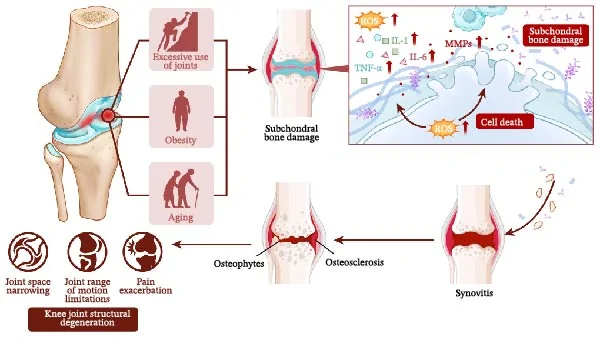
Pathological mechanisms and structural changes in knee joint degeneration, highlighting the impact of aging, obesity, and excessive joint use on subchondral bone damage and associated cellular responses.

Osteoarthritis (OA) is the most common type of arthritis and the major form of age‐related degenerative joint disease. It is characterized by progressive degeneration of the articular cartilage and pathological changes in the surrounding tissues. With the global population aging, the prevalence and incidence of OA have continued to increase, making it a major cause of pain and disability among middle‐aged and elderly adults (Figure 1). This imposes a substantial economic and psychological burden on patients and health systems across the globe [1].

**Figure 2:**
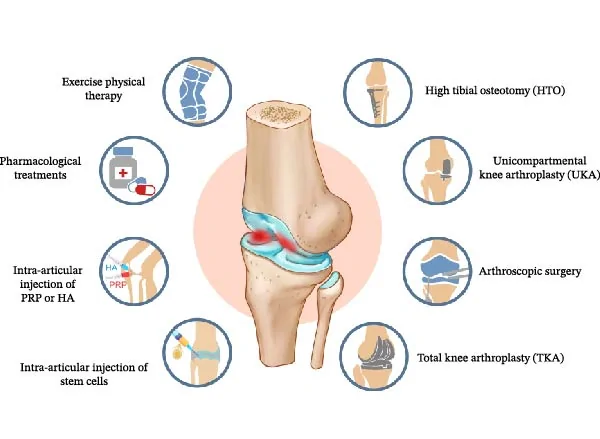
Therapeutic modalities of knee joint disease: conservative management, pharmacological therapy, intra‐articular drug, and arthroscopic or replacement surgery [1–3].

Currently available OA therapies consist of medications, intra‐articular injections, and surgery. Nonetheless, all of these modalities have certain limitations. For example, over‐the‐counter nonsteroidal anti‐inflammatory drugs (NSAIDs) are routinely employed for pain relief and anti‐inflammatory purposes; however, they cannot repair the joint structures, and long‐term exposure to them has been reported to increase the incidence of gastrointestinal events and cardiovascular risk, usually in a dose‐dependent fashion. Symptomatic slow‐acting drugs, such as glucosamine and chondroitin sulfate, have shown promise for reducing clinical disease progression, but their time to efficacy is long, and they do not meaningfully restore damaged structure. Likewise, injections of corticosteroids, hyaluronic acid (HA), or platelet‐rich plasma (PRP) into the joint space may provide only temporary relief, and the long‐term efficacy and safety of both pharmacotherapy and injection therapy remain uncertain [2]. The surgical alternatives are predominantly indicated for end‐stage disease. However, although these procedures may alleviate severe symptoms, they are major surgeries that entail substantial cost as well as risks such as infection, bleeding, or thromboembolic events. Prosthetic implants also have a limited functional lifetime that restricts their application to younger or more active patients. Importantly, none of them can completely reverse cartilage loss or stop the development of OA, and more novel treatments are certainly needed that, in addition to being both safe and efficient, address the disease at its source, i.e., by restoring the damaged joint microstructure (Figure 2).

Recently, stromal vascular fraction gel (SVF)‐gel has attracted increasing interest as a therapeutic strategy in tissue engineering and may provide a novel approach for treating OA. SVF‐gel is a heterogeneous, cell‐rich matrix obtained by mechanical or enzymatic dissociation of adipose tissue and contains mesenchymal stem cells (MSCs), endothelial progenitor cells (EPCs), multiple populations of immune cells, and a series of biologically relevant molecules. These components include anti‐inflammatory cytokines such as interleukin‐10 (IL‐10) and transforming growth factor‐β (TGF‐β), regulators of tissue repair, and antagonists of inflammatory cascades such as IL‐1 receptor antagonist (IL‐1Ra) [3, 4]. SVF‐gel also contains pivotal pro‐repair factors, including vascular endothelial growth factor (VEGF), hepatocyte growth factor (HGF), and platelet‐derived growth factor (PDGF) [5]. All these components contribute to the regenerative effects of SVF‐gel, including cell activation, promotion of tissue regeneration, modulation of the local inflammatory response, inhibition of inflammation, and neovascularization, which may make SVF‐gel a promising biological material to address the complex pathology of OA, especially cartilage degeneration and chronic synovitis. Compared with other cell‐based or biologically derived modalities, such as PRP or isolated MSCs, SVF‐gel stands out because of its composite and synergistic structure. Due to its multiphasic and broad biological activities, SVF‐gel offers the potential for use in the field of regenerative orthopedics [6].

In this review, we focus on the therapeutic rationale, biological functions, and potential mechanisms of SVF‐gel in OA, together with the current progress in preclinical studies, emerging clinical evidence, combination strategies, and translational challenges. By emphasizing how the biological properties of SVF‐gel may address key pathological features of OA and how these effects may translate into patient‐centered benefits, we aim to clarify its current therapeutic potential and future prospects in regenerative treatment.

## 2. Pathophysiological Background and Therapeutic Challenges of OA

### 2.1. Pathogenesis of OA

#### 2.1.1. Chondrocyte Apoptosis and Extracellular Matrix (ECM) Degradation

OA is a chronic joint disease characterized by progressive cartilage degeneration and persistent low‐grade inflammation. Its development is driven by multiple interacting factors, including mechanical stress, inflammatory signaling, and disruption of joint homeostasis. Among the most important pathological features are chondrocyte apoptosis and ECM degradation, which together accelerate structural damage and functional decline of the joint [7, 8].

Two of the major pathological attributes of OA are aberrant chondrocyte apoptosis and abnormal chondrocyte differentiation [9]. In OA, chondrocyte apoptosis outpaces cell survival, disrupting the homeostasis. As a result of this, large numbers of chondrocytes are lost and can no longer perform their normal functions. Consequently, the ECM is severely damaged, particularly in key components such as type II collagen and proteoglycans, and the weakened cartilage can no longer effectively cushion the joints under load, thereby accelerating disease progression.

Articular cartilage represents an unusual connective tissue that is devoid of a vascular and nerve supply, and its role is to provide structural support to joints and to facilitate movement without friction by creating a layer of lubrication. It has adequate resilience and durability given the presence of its ECM, composed of type II collagen fibers, aggrecan, and water‐storing macromolecules. These matrix elements together confer biomechanical functionality to the cartilage, such as compressive stiffness, fluid retention, and lubrication, which are of utmost importance for mechanical load‐bearing [10].

Articular cartilage is mainly composed of hyaline cartilage, of which there are two distinct types: transient and permanent [11]. Permanent cartilage in healthy joints is in a steady state, where the cellular phenotype is one of very limited proliferation and no terminal differentiation. Under OA conditions, this homeostasis is disrupted. The chondrocytes in permanent cartilage start to lose the phenotype of hyaline cartilage and exhibit features of endochondral ossification. This degenerative progression is characterized by the increase of proliferation, hypertrophy, and aberrant differentiation [12]. This is associated with ECM mineralization, cartilaginous calcification, and vascular invasion, which accelerate the degradation and deterioration of joint function. With time, the cartilage becomes calcified and sclerotic, which involves a decrease in its elasticity and consequently in the mechanical function [13]. These structural changes can explain the generation of osteophytes, which are currently acknowledged as the characteristic pathological feature of the progression of OA.

#### 2.1.2. Synovitis and Its Role in OA Progression

The synovium contributes to joint function through the secretion of synovial fluid, which lubricates and protects articular surfaces. Synovial fluid contains vital molecules, such as lubricin and HA, that reduce friction and prevent protein accumulation [14]. The synovium is frequently inflamed in OA, termed synovitis, which is characterized by inflammatory cell infiltration, synovial hyperplasia, and reduced secretion of lubricin and HA. These changes impair synovial fluid physiology and facilitate cartilage deterioration [15]. Synovitis occurs across all phases of OA and correlates highly with the pain severity and structural damage of the joint. During synovitis, inflammatory mediators cause nociceptive nerve endings to sensitize and hence promote pain in a chronic condition. Increasing evidence indicates that the severity of synovitis may predict the progression of OA, and it is proposed to be a valuable treatment target [16]. In addition, aberrant angiogenesis in the synovium and subchondral bone has been increasingly recognized as a pathological feature of OA. Unlike controlled reparative vascular remodeling, excessive or ectopic neovascularization may contribute to osteophyte formation, cartilage calcification, inflammatory cell infiltration, and pain sensitization, thereby further promoting the disease progression.

#### 2.1.3. Oxidative Stress and Joint Microenvironment Dysregulation

Repetitive mechanical stress, obesity, diabetes, and metabolic syndrome can disrupt the intra‐articular microenvironment and contribute to the onset and progression of OA [17]. A central mechanism involved is the excessive generation of reactive oxygen species (ROS). While physiologically moderate levels of ROS function as signaling molecules and help maintain the redox balance, pathological accumulation in OA induces oxidative stress and overwhelms the cellular antioxidant defenses. This redox imbalance results in oxidative damage to DNA, proteins, and lipids, impairing cellular function and activating inflammatory pathways [18]. Higher ROS levels also contribute to cartilage breakdown by inducing chondrocyte apoptosis, inhibiting ECM synthesis, and stimulating matrix‐degrading molecules, including matrix metalloproteinases (MMPs) [19]. These processes all contribute to cartilage breakdown, enhance synovial inflammation, and accelerate OA progression.

#### 2.1.4. Imbalance of Pro‐ and Anti‐Inflammatory Factors

An important pathological mechanism of OA is the imbalance between pro‐ and anti‐inflammatory mediators, leading to joint dysregulation and accelerating disease progression [20]. This imbalance is characterized by the increased activity of MMPs, proteolytic enzymes that play a central role in ECM turnover. One major target is type II collagen, an essential matrix component. The loss of type II collagen impairs both the cartilage structure and its protective barrier function, thereby increasing the risk of further joint damage [21]. In addition to structural weakening, the MMP‐dependent degradation of the ECM during OA results in the release of molecular fragments such as DAMPs that are recognized by synovial fibroblasts and macrophages, resulting in the secretion of proinflammatory cytokines that exacerbate synovitis and destruction of cartilage [22].

IL‐1β, tumor necrosis factor (TNF‐α), and IL‐6 are pro‐inflammatory cytokines that contribute to the pathogenesis of OA by promoting dysregulation of ECM homeostasis as well as maintaining chronic inflammation. IL‐1β can negatively regulate the genes responsible for type II collagen and proteoglycan synthesis, consequently harming their deposition. Moreover, it can stimulate pathways such as ERK, which also activate catabolic factors such as collagenases and aggrecanases, leading to the degradation of the cartilage matrix. The ultimate result is chondrocyte hypertrophy, dedifferentiation, and death [23]. TNF‐α contributes to the catabolic cascade by augmenting the actions of IL‐1β and directly down‐regulates ECM synthesis [24]. IL‐6 is elevated in the synovial fluid of OA patients and positively correlates with pain severity, suggesting that the mediator is associated with inflammation and symptom expression [25].

Together, these cytokines set off a vicious cycle by inducing catabolic cascades, disrupting the balance between ECM production and destruction, as well as perpetuating synovial inflammation, thus constituting a cascading, degenerative process in OA.

### 2.2. Current Status and Limitations of OA Treatment

At present, OA management is guided by a stepwise treatment protocol including physical therapy, pharmacological agents, mainly NSAIDs, intra‐articular injections such as HA, PRP, and MSCs, and surgical interventions such as osteotomy, arthroscopy, and total joint replacement. Crucially, none of these interventions can reverse the hallmark pathology of OA, namely, cartilage degeneration, and therefore are unlikely to be able to counteract the pathophysiology of the disease directly.

Exercise and other conservative treatments are commonly recommended, especially in early‐stage OA, because they can improve muscle strength, joint mobility, and symptom control. However, their benefits are often limited in moderate‐to‐severe OA and are strongly influenced by long‐term patient adherence.

NSAIDs remain widely used for symptomatic relief because of their analgesic and anti‐inflammatory effects, while supplements such as glucosamine and chondroitin sulfate have also been applied to support joint health. Nevertheless, long‐term NSAID use is associated with safety concerns, and the clinical efficacy of these supplements remains controversial [26, 27].

Support for cartilage synthesis and improved mechanical behavior has also been attributed to glucosamine and chondroitin sulfate, two commonly used supplements for OA. Although their efficacy remains controversial, some studies have indicated that their effects are similar to those of the placebo. In addition to a few cases of nausea and diarrhea as adverse effects, glucosamine and chondroitin sulfate are generally not accepted in clinical practice [28, 29].

Intra‐articular injection is often employed as a treatment modality for moderate to severe OA. HA, PRP, and MSCs are among the most commonly injected agents. HA injection is intended to restore joint lubrication and relieve symptoms; however, the therapeutic effect is transient, lasting for several months, and the long‐term benefits of such treatment for cartilage protection remain controversial [30]. PRP contains a highly concentrated mixture of autologous growth factors. There is evidence that PRP has the potential to contribute to tissue regeneration and symptom relief. Nevertheless, the efficacy and safety of PRP have not yet been fully validated by large randomized controlled trials (RCTs) [31, 32], whereas MSC therapy is a promising option for cartilage regeneration through differentiation and paracrine effects. However, a variety of obstacles still impede their use in a clinical setting, namely, immune response and donor heterogeneity risk, high manufacturing costs, and an incomplete understanding of their biological mechanisms. Some adverse effects such as joint swelling, inflammation, and anti‐histone antibody production have also been reported [33–35]. In sum, though intra‐articular injections provide some acute relief of pain, in some circumstances or in specific patients, they cannot be considered capable of arresting or reversing the process of articular cartilage degeneration.

**Figure 3:**
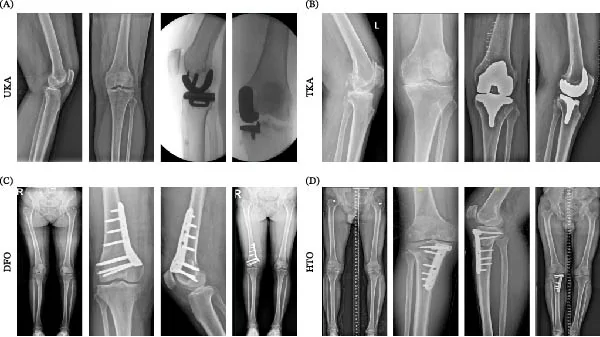
Radiographic presentations of various surgical approaches for OA. (A) Unicompartmental knee arthroplasty (UKA); (B) total knee arthroplasty (TKA); (C) distal femoral osteotomy (DFO); and (D) high tibial osteotomy (HTO). All radiographic images were obtained from the Orthopedic Medical Center, Linyi People’s Hospital.

In most cases, surgery is only indicated for patients with moderate to severe OA who fail conservative treatment to relieve symptoms or normalize joint function (Figure 3). Typical surgical treatments include osteotomy, arthroscopy, and total joint replacement surgery. For appropriate patients, such as those with specific joint deformities, osteotomy may be employed to alleviate pain by redistributing mechanical loads through alignment of the axis of weight‐bearing. However, its use is restricted by rigorous patient selection criteria and inconsistent results [36]. Arthroscopy removes intra‐articular debris and reshapes torn cartilage or the synovial pathology. Despite its minimally invasive nature, evidence suggests that arthroscopy provides, at best, only short‐term symptom relief and is not found to provide superior results to those obtained with nonsurgical intervention. For example, patients undergoing arthroscopic surgery for degenerative meniscal tears have been three times more likely to undergo joint replacement than those receiving physical therapy alone [37–39]. In end‐stage OA, total joint replacement is the gold standard of therapy and is associated with an improvement in pain, mobility, and quality of life. Nevertheless, implantation of prostheses has an average lifetime of 15–20 years [40], which would be a source of concern for younger patients, who will most likely undergo revision operations later in life. In addition, all surgeries are associated with complications such as postoperative bleeding, infection, and venous thromboembolism. Indeed, the overall incidence of venous thrombosis without prophylaxis may be estimated as high as up to 50% [41].

Although a number of treatment options exist, none can restore the loss of tissue due to damage. Because the treatments for OA do not correct the root pathology—cartilage degeneration, treatment of OA will inevitably continue to be a challenge because curing the disease may require addressing the destruction of cartilage. Both pharmacological and surgical treatments are associated with risks and complications, particularly in patients at higher ages. There are numerous surgical and nonsurgical options that provide only temporary pain relief, but none directly addresses the underlying cartilage degeneration in OA. This situation challenges the approach to pain management of patients suffering from OA, especially as opioid‐based treatments are now contraindicated due to the opioid epidemic. Moreover, cutting‐edge therapies, including injections of MSCs and total joint replacement, are extremely expensive and therefore not accessible to low‐ and middle‐income countries.

### 2.3. Theoretical Basis of SVF in OA Treatment

SVF is a heterogeneous cell population derived from adipose tissue that has attracted attention due to its distinct composition and functionality. SVF is a mixture of different cell types, such as MSCs, EPCs, and immunoregulatory cells, which effectively work together for tissue regeneration, immunomodulation, and angiogenesis [42]. Consequently, SVF is a promising candidate for the regenerative treatment of OA (Table 1).

**Table 1:** Main components and putative functions in SVF‐gel.

Cell Type	Mainly secreted factors	Function
Mesenchymal stem cells (MSC)	Vascular endothelial growth factor (VEGF) [10] Hepatocyte growth factor (HGF) [10] Transforming growth factor‐β (TGF‐β) [11] Platelet‐derived growth factor (PDGF) [12] Interleukin‐6 (IL‐6) Insulin‐like growth factor‐1 (IGF‐1) [12] Tumor necrosis factor‐stimulated gene‐6 (TSG‐6) Basic fibroblast growth factor (FGF‐2) [12]	(1) Promotes neovascularization and improves local blood flow [13] (2) Anti‐fibrosis, promoting cell migration and regeneration [14] (3) Regulating immune and inflammatory responses, promoting ECM synthesis, and reducing tissue damage [15] (4) Regulating the ratio of matrix metalloproteinase (MMPs) and their tissue inhibitory factors (TIMPs) and inhibiting excessive protease activity within the ECM [14]
Endothelial progenitor cells (EPCs)	Vascular endothelial growth factor (VEGF) Angiopoietin‐1/2 Nitric oxide (NO) Platelet‐derived growth factor (PDGF) Insulin‐like growth factor‐1 (IGF‐1)	(1) Promotes blood vessel formation and improves local blood flow [13] (2) Recruitment of pericytes and stabilization of neovascularization [13]
Pericyte	Vascular endothelial growth factor (VEGF) Transforming growth factor‐β (TGF‐β)	(1) Supports vascular endothelial cells and blood vessel formation [13] (2) Promotes vascular maturation and stabilization [12]
Macrophages (M2)	Interleukin‐10 (IL‐10) Transforming growth factor‐β (TGF‐β)	(1) Suppresses inflammation and promotes tissue repair; Promotes cell regeneration and fibrosis repair [16]
Lymphocytes (Treg)	Interleukin‐10 (IL‐10) Interleukin‐4 (IL‐4) Interleukin‐13 (IL‐13)	(1) Suppression of excessive immune responses [14] (2) Promotes M2‐type macrophage transformation and reduces inflammation [17]

*Note:* The functions listed in this table summarize the reported or putative roles of individual SVF‐gel components. However, the therapeutic effects of intact SVF‐gel may not be attributable to any single component alone. Direct comparative studies using isolated MSCs, EPCs, immune‐cell populations, SVF cells without ECM support, ECM‐only or decellularized matrices, conditioned medium or extracellular vesicles, and intact SVF‐gel are needed to clarify the relative contribution of each component and their potential synergistic interactions.

Among the various cell types, MSCs are among the most important components of SVF due to their multipotent differentiation capacity. MSCs can differentiate into chondrocytes, vascular endothelial cells, smooth muscle cells, and pericytes, which are important for cartilage regeneration and vascular regeneration [43]. Many studies have demonstrated that MSCs control the vascular growth, maturation, and stabilization via several signaling pathways, such as inducing cartilage differentiation and healing by secreting TGF‐β, aiding vascular remodeling via Ang‐2, and stabilizing vascular maturation by secreting PDGF‐B [44], all of which reflect the pivotal role played by MSCs in repairing and regenerating tissue, making them attractive therapeutic candidates for OA therapy.

EPCs, also a significant fraction of the SVF, are involved in angiogenesis and repair processes. EPCs are able to synthesize angiogenic factors, including VEGF and insulin‐like growth factor‐1 (IGF‐1), which trigger angiogenesis and vascularization and contribute to the repair of damaged tissues. EPCs can also act together with MSCs to effectively trigger angiogenesis and form vascularized tissue. This interaction greatly promotes local blood circulation, thereby creating a favorable microenvironment for cartilage repair and regeneration [45].

Immunoregulatory cells are an essential component of SVF and mediate various biological activities. Macrophages account for a substantial proportion of the cellular components in the SVF. Almost all macrophages in SVF are of M2 phenotype, which are mainly associated with anti‐inflammatory function and mediate against inflammation by releasing anti‐inflammatory cytokines, including IL‐4, IL‐10, and TGF‐β. They also secrete pro‐angiogenic molecules such as VEGF, important for forming a vascular network as well as wound repair [46]. T cells participate as well, maintaining immune homeostasis by working with the other cells to bring about healing of tissues. They can promote macrophage polarization toward the M2 phenotype, forming a positive feedback loop promoting the anti‐inflammatory response and functional recovery.

In view of the complex composition and multi‐functionality of SVF, its constituent cells may work together to exert synergistic effects in tissue regeneration and functional recovery. For example, MSCs and EPCs may cooperate to regulate vascular remodeling and provide trophic support for tissue repair, whereas immunoregulatory cells may suppress excessive inflammation and create a favorable healing microenvironment [47]. In addition, the ECM retained in the SVF‐gel may provide a three‐dimensional (3D) scaffold that supports cell survival, local retention, and sustained paracrine signaling [48]. However, it remains unclear whether the therapeutic effects of SVF‐gel are primarily attributable to individual components, such as MSCs or ECM, or to synergistic interactions among multiple cellular and matrix components. Therefore, direct comparative studies evaluating intact SVF‐gel against isolated MSCs, SVF cells without matrix support, ECM‐only or decellularized matrices, and conditioned medium are needed to clarify the unique therapeutic advantages of SVF‐gel in OA treatment.

## 3. Preparation, Characteristics, and Biological Functions of SVF‐Gel

### 3.1. Preparation Methods of SVF‐Gel

SVF‐gel is a semisolid biomaterial derived from adipose tissue with high elasticity and excellent biocompatibility. Adipose tissue is typically harvested from the abdominal or gluteal region. Adipose tissue can be processed using enzymatic digestion and/or mechanical methods to extract SVF [49].

**Figure 4:**
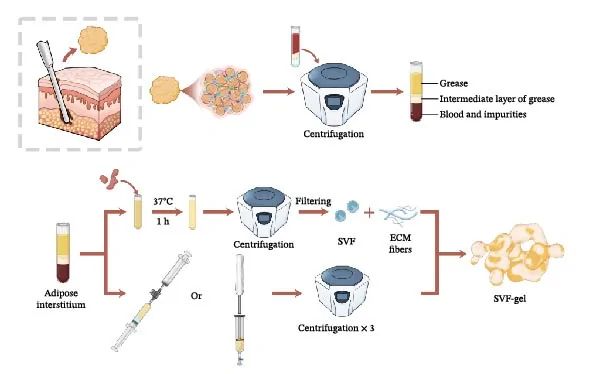
Schematic representation of the preparation process for SVF‐gel [4–6].

Enzymatic digestion is the technique in which the adipose tissue is treated with an appropriate amount of type I collagenase to digest all ECM components of the adipose tissue (Figure 4). In enzymatic protocols, lipoaspirate is typically digested with collagenase under controlled temperature and agitation, followed by neutralization of enzyme activity using a serum‐containing medium and subsequent centrifugation to isolate the SVF cell fraction [50]. Importantly, the exact conditions vary among studies, including the collagenase concentration, tissue‐to‐enzyme ratio, digestion time, centrifugation parameters, and the type of serum‐containing medium used for neutralization. In experimental settings, fetal bovine serum (FBS)‐containing medium is commonly used, whereas clinically compliant protocols may require xeno‐free or otherwise standardized alternatives. These isolated SVF cells are then suspended in phosphate‐buffered saline (PBS) and passed through a cell strainer to eliminate undesirable, undigested tissue fragments [45, 51]. Once purified, the SVF cells are mixed with a scaffold material at an appropriate ratio to form an elastic and bioactive gel.

Another method to generate SVF involves mechanical fractionation, which can be performed using the physical force of breaking the tissue. The process involves ultrasonic emulsification, shearing, or other mechanical forces to fractionate the tissue, followed by three cycles of centrifugation to isolate the cellular components, ultimately yielding a semifluid gel‐like material (Table 2). Mechanical separation, compared with enzymatic digestion, presents the following benefits: the procedure is less complex, enzymatic reagents are omitted, it is more time‐ and cost‐effective, higher integrity of the ECM is kept, and there is a lower probability of immune reactions related to residual enzymes [48]. Yet, enzymatic digestion is used in cases that have as a prerequisite higher cell yield of SVF cells and a higher proportion of progenitor cells [52].

**Table 2:** Comparison of mechanical versus enzymatic isolation of SVF‐gel.

Preparation method	Mechanical isolation	Enzymatic isolation
Cell yield [18]	0.3 × 10^4^–26.7 × 10^5^/mL	2.3 × 10^5^–18 × 10^5^/mL
Cell composition [18, 19]	Endothelial progenitor cells (3%–5%) Stem cells (40%–45%)	Endothelial progenitor cells (10%–12%) Stem cells (24%–28%)
Safety [18]	High, no exogenous enzymes	Need to consider enzyme safety
Operation process [20, 21]	Simpler and easier to obtain	Strict, higher requirements
Operation time [18, 20]	8–20 min	50–210 min
Operating cost [22]	Low, no expensive consumables	High
Extracellular matrix (ECM) integrity [22, 23]	Intact	Partially damaged
Application scenarios [24]	Suitable for large‐scale promotion	Suitable for laboratory validation

SVF‐gel is isolated from adipose tissue harvested from the same patient; it demonstrates remarkable biocompatibility, biodegradability, low risk of immune rejection, and lack of toxic degradation byproducts. Therefore, SVF‐gel is a candidate for a safe and effective biomaterial for therapy. Because this article is a narrative review rather than a methodological report, we summarize the general preparation workflow of SVF‐gel rather than proposing a single standardized protocol. At present, no universally accepted protocol for SVF‐gel preparation has been established. Overall, key procedural variables, including tissue source, processing method, enzyme exposure, neutralization conditions, and centrifugation settings, remain inconsistent across studies, highlighting the need for further methodological standardization.

### 3.2. Biological Functions of SVF‐Gel in OA

#### 3.2.1. Anti‐Inflammatory Effects: Regulation of Immune Responses and Inhibition of Inflammatory Factors

**Figure 5:**
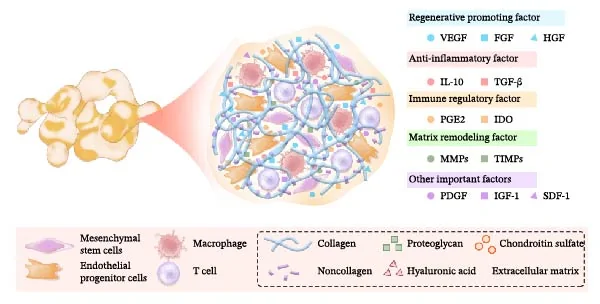
Key cells, cytokines, and proteins present in SVF‐gel, including regenerative promoting factors, anti‐inflammatory factors, immune regulatory factors, matrix remodeling factors, and extracellular matrix components [7–9].

SVF‐gel is rich in MSCs and macrophages. More than 90% of the macrophages are M2‐type, releasing anti‐inflammatory cytokines such as IL‐4, IL‐10, and TGF‐β to suppress the inflammation and facilitate tissue repair (Figure 5). MSCs present in SVF‐gel regulate the inflammation through the release of anti‐inflammatory cytokines. The immunomodulatory function of MSCs is also illustrated by their ability to convert macrophages from a pro‐inflammatory M1 phenotype to an anti‐inflammatory M2 phenotype, and this effect may markedly downregulate synovial inflammation. Since synovitis is an important pathological characteristic of OA, its inhibition can sufficiently abrogate the cascade of cartilage destruction by inflammatory mediators and ultimately prevent the progression of OA [53].

In addition, the MSCs prevent over‐activation of T cells, which relieves the pro‐inflammatory condition, meanwhile inducing the expansion of regulatory T cells (Tregs), a subset of T cells endowed with immunoregulatory functions that help maintain immune homeostasis and boost M2 macrophage activity [54]. These two links form a positive feedback loop in the inflammatory microenvironment, which can significantly relieve the inflammatory response.

In addition to these cellular interactions, several intracellular signaling pathways and soluble mediators may participate in the immunomodulatory activity of the MSC‐containing SVF‐gel. MSCs and related stromal cell populations can regulate macrophage phenotype mainly through paracrine mediators, including prostaglandin E2 (PGE2), TGF‐β, IL‐10, indoleamine 2,3‐dioxygenase, and extracellular vesicle‐associated microRNAs. Recent single‐cell evidence indicates that mechanical activation of stem cell populations by microgel carriers can promote Yes‐associated protein (YAP) nuclear translocation and induce an immunoregulatory Pdpn+Grem1+Ptgs2+ stromal cell phenotype. Activation of the YAP/PTGS2/PGE2 axis suppresses macrophage expression of TNF‐α and IL‐1β while increasing IL‐10 expression, thereby promoting the transition from a pro‐inflammatory M1‐like phenotype toward an anti‐inflammatory M2‐like phenotype [55]. This mechanism may be particularly relevant to the SVF‐gel because its 3D matrix and mechanical microenvironment may influence stromal cell behavior and paracrine activity. Moreover, MSC‐derived secretomes and extracellular vesicles contain OA‐relevant miRNAs, including miR‐24‐3p, miR‐125b‐5p, and miR‐222‐3p, which are associated with anti‐inflammatory, matrix‐regulatory, and chondroprotective effects [56]. These mediators may converge on inflammatory pathways such as NF‐κB signaling, resulting in reduced production of IL‐1β, TNF‐α, IL‐6, MMPs, and other catabolic factors. Therefore, the anti‐inflammatory effects of SVF‐gel may depend not only on the presence of M2 macrophages and MSCs but also on coordinated regulation of YAP/PTGS2/PGE2 signaling, EV‐miRNA‐mediated paracrine communication, and suppression of pro‐inflammatory intracellular pathways.

#### 3.2.2. Promoting Cartilage Repair: Enhancing Chondrocyte Proliferation and Differentiation

MSCs in the SVF‐gel are enriched for cells with specific markers and the ability to differentiate into chondrocytes. Evidence suggests that MSCs also secrete high levels of paracrine cytokines and regenerative factors, such as HGF and IGF‐1, which stimulate tissue repair by promoting chondrocyte proliferation and activity. Under specific microenvironmental conditions, such as hypoxia, mechanical stimulation, and growth factor induction, SVF‐gel MSCs upregulate cartilage‐specific genes encoding type II collagen and proteoglycans, both of which are involved in cartilage repair and regeneration [57]. MSCs also play an important role in balancing ECM components and metabolism. A balance between MMPs and tissue inhibitors of metalloproteinases (TIMPs) is maintained, thus avoiding any extreme ECM degradation. MSCs specifically downregulate MMPs such as MMP‐13 as well as a disintegrin and metalloproteinase with thrombospondin motifs 5 (ADAMTS‐5) enzymes, which are major players in cartilage breakdown [58], while upregulating the expression of type II collagen and proteoglycan, which promotes cartilage restoration and maintains its structural integrity [59].

At the intracellular signaling level, the chondroprotective and cartilage‐reparative effects of MSC‐containing SVF‐gel may involve multiple OA‐related pathways. The TGF‐β/Smad pathway is one of the central regulators of chondrogenic differentiation, facilitating the upregulation of SOX9 expression and consequently enhancing the transcription of cartilage matrix genes such as COL2A1 and ACAN. In contrast, dysregulated Wnt/β‐catenin signaling is closely associated with OA progression and interacts with several other pathways, including TGF‐β, BMP, Indian Hedgehog, Notch, NF‐κB, and FGF signaling [60]. Excessive activation of β‐catenin may promote chondrocyte hypertrophy, matrix catabolism, and the expression of MMP‐13 and ADAMTS‐5, whereas appropriate modulation of this pathway may help maintain cartilage homeostasis. In addition to classical chondrogenic pathways, emerging studies suggest that cellular senescence and ferroptosis are important mechanisms influencing cartilage repair in the OA microenvironment [61]. For example, miR‐24 has been shown to attenuate cellular senescence and promote chondrogenesis by targeting TAOK1 in synovial MSC organoids. Concurrently, miR‐140‐3p has the capacity to modulate inflammation, oxidative stress, and ferroptosis by targeting the cartilage intermediate layer protein [62, 63]. These findings suggest that MSC‐derived paracrine factors and extracellular vesicle‐associated miRNAs may contribute to cartilage protection by regulating senescence, mitochondrial metabolism, glycolysis/OXPHOS balance, oxidative stress, and ferroptotic cell death. Accordingly, SVF‐gel may support cartilage repair through an integrated mechanism involving TGF‐β/Smad‐SOX9‐mediated matrix synthesis, modulation of Wnt/β‐catenin‐related catabolic signaling, inhibition of inflammatory NF‐κB activity, and attenuation of senescence‐ and ferroptosis‐associated cartilage degeneration.

Moreover, SVF‐gel is incorporated with ECM, including collagen and HA, as the 3D scaffolding component for restoring the cartilage tissue, which supports cell adhesion, growth, and differentiation, thus forming a microenvironment conducive to therapeutic cartilage regeneration [64]. These modes of action together support a highly promising biomaterial of SVF‐gel for stimulating cartilage repair and ameliorating progression of OA.

#### 3.2.3. Improving Local Microcirculation: A Context‐Dependent Role of Angiogenesis

SVF‐gel provides a heterogeneous population of stromal cells, including adipose‐derived stem cells, EPCs, pericytes, and macrophages, which may contribute to vascular remodeling through both direct and indirect mechanisms that support tissue repair and regeneration [58]. HGF and VEGF secreted by the adipose‐derived stem cells, together with angiogenic cytokines released by EPCs, can promote endothelial cell proliferation, migration, and lumen formation [65]. EPCs also interact with pericytes, which help stabilize newly formed vessels and maintain vascular integrity [66]. Through these coordinated effects, SVF‐gel may improve local perfusion and provide a microenvironment with enhanced oxygen and nutrient supply, thereby supporting tissue repair.

However, the role of angiogenesis in OA should be interpreted cautiously. In OA, aberrant angiogenesis in the synovium and subchondral bone has been associated with osteophyte formation, cartilage calcification, synovitis progression, and pain sensitization [67]. Therefore, the potential benefit of SVF‐gel is unlikely to arise from indiscriminate stimulation of neovascularization. Instead, its vascular‐related effects may depend on promoting controlled and context‐appropriate microvascular remodeling, improving the periarticular microenvironment without exacerbating pathological vascular invasion into inappropriate regions. In this sense, SVF‐gel may function more as a modulator of vascular homeostasis than as a simple pro‐angiogenic agent. Future studies are needed to clarify the stage‐specific, dose‐dependent, and site‐specific vascular effects of SVF‐gel in OA.

#### 3.2.4. Improving the Joint Cavity Microenvironment: Unique 3D Structure and Enhanced Matrix Metabolism

Due to the gel‐like matrix and multi‐component property, SVF‐gel helps to provide a better micro‐environment in the joint cavity, thus improving therapeutic outcomes in patients with OA. The 3D matrix intrinsic to SVF‐gel can effectively support cell adhesion and proliferation, thereby extending cell viability and survival time within the joint cavity. Its matrix structure can ensure the slow release of growth factors or cytokines so as to achieve a long‐term therapeutic effect. Further, SVF‐gel itself enhances lubricating features of joints and, as a result, decreases mechanical damage to the cartilage. Compared with conventional hyaluronan‐based gels, SVF‐gel may provide enhanced joint lubrication because of its biological activity and regenerative potential.

Oxidative stress plays a crucial role in OA‐induced chondrocyte death, and thus, antioxidant SVF‐gel can significantly diminish this kind of chondrocyte apoptosis. SVF‐gel can reduce ROS overaccumulation to exert an antioxidant role on the chondrocytes and thus keep the viability of the chondrocytes and enhance the cartilage survival. Additionally, SVF‐gel can regulate the matrix metabolism by regulating ECM synthesis and degradation for restraining cartilage degeneration and thus inhibit the OA progression to protect joint function [64].

## 4. Progress in Animal Experiments and Clinical Studies

The key methodological and outcome characteristics of representative preclinical and clinical studies related to SVF/SVF‐gel‐based therapies are summarized in Table 3.

**Table 3:** Synopsis of representative preclinical and clinical evidence for SVF/SVF‐gel‐based therapies.

Evidence type	Disease/model	Study design	Sample size	Intervention/comparator	Follow‐up	Main outcomes
Preclinical [25]	OA	Animal experimental study	32 rabbits	Compared with MSCs	1 month	Osteochondral repair; reduction of synovial reactions; reduction of degenerative changes in the meniscus
Preclinical [26]	OA	Animal experimental study	30 rats	Compared with NS and MSCs	14 days	Articular surface appeared smooth, accompanied by reduced levels of inflammatory cytokines and type II collagenase
Preclinical [27]	OA	Animal experimental study	36 rats	Compared with HA and MSCs	8 weeks	Promote cartilage matrix synthesis, inhibit hypertrophic differentiation, and improve bone structure
Preclinical [28]	Abdominal wall hernia	Animal experimental study	28 immunodeficient rats	Comparison of SVF‐coated mesh with acellular fibrin‐coated mesh	21 days	Well tolerated and accelerated maturation of the vascular network
Clinical, OA [29]	OA	Meta‐analysis	22 studies, >3000 patients	Compared with HA and MSCs	3–36 months	Significant improvement of VAS, WOMAC, and ROM in SVF group
Clinical, non‐OA [30]	Keloids and hypertrophic scars	Meta‐analysis	20 studies, 493 patients	Compared with MSCs	3–24 months	Significant improvements in both the POSAS and VSS, scar surface area and thickness were significantly decreased
Clinical, non‐OA [31]	Androgenic alopecia	Prospective clinical studies	30 patients	SVF‐based therapy	6 months	Hair shaft diameter increased by 51.35%, while hair density improved by 16.09%
Clinical, non‐OA [32]	Unilateral vocal fold paralysis	Retrospective study	22 patients	SVF‐based therapy	18 months	GRBAS evaluation results showed that the voice quality improved significantly at 12 months and remained stable at 18 months after the operation
Clinical, non‐OA [33]	Striae distensae	Prospective clinical studies	36 patients	SVF‐based therapy	6 months	GAIS score was 1.87 ± 0.03, the depth, area, and color of SD improved
Clinical, non‐OA [34]	Hollowing temples	Prospective clinical studies	63 patients	Compared with Coleman’s fat	12 months	The SVF‐gel group showed more pronounced temporal contour augmentation and higher patient satisfaction

### 4.1. Animal Studies

Accumulating evidence from animal studies supports the anti‐inflammatory and cartilage‐protective properties of SVF‐gel. In a surgically induced OA model established by anterior cruciate ligament transection, intra‐articular administration of SVF‐gel was compared with other commonly used injectables, including HA, MSCs, and conventional SVF. Methodologically, this type of model induces joint instability and progressive cartilage degeneration, thereby allowing the evaluation of both synovial inflammation and cartilage structural damage. After intra‐articular treatment, SVF‐gel produced notable improvements in synovial pathology, including a smoother synovial lining, reduced sub‐synovial hypertrophy, and diminished vascularization [68]. At the molecular level, SVF‐gel enhanced the expression of chondrogenic markers such as COL2, SOX9, and ACAN while reducing COL10 expression, suggesting attenuation of chondrocyte hypertrophy and OA progression [69–71]. Histological evaluation further confirmed a significant reduction in OA‐associated degenerative changes. Compared with conventional SVF, SVF‐gel may provide additional therapeutic advantages owing to its native ECM‐rich and gel‐like architecture. This jelly‐like structure may improve intra‐articular retention, support sustained release of growth factors, and provide a 3D microenvironment for cell survival, paracrine signaling, and tissue repair. These properties may explain why SVF‐gel appears to exert stronger anti‐inflammatory and cartilage‐reparative effects than SVF alone in experimental settings [70]. Collectively, these preclinical findings suggest that the SVF‐gel may alleviate synovial inflammation, inhibit cartilage degeneration, and promote cartilage matrix restoration in OA models.

However, the interpretation of animal studies should be cautious because commonly used OA models do not fully reproduce the chronic and progressive nature of human OA. Chemically induced models, such as the monosodium iodoacetate (MIA) model, mainly induce rapid cartilage degeneration through chondrocyte metabolic disruption and cytotoxicity, whereas human OA is usually driven by long‐term interactions among low‐grade inflammation, mechanical stress, aging, metabolic dysregulation, synovitis, and subchondral bone remodeling. Surgically induced models, such as destabilization of the medial meniscus (DMM), anterior cruciate ligament transection, or meniscectomy, are useful for studying post‐traumatic or mechanically unstable OA, but they often progress more rapidly than primary human OA and may not fully reflect age‐related or obesity‐associated disease phenotypes. In addition, differences in joint size, cartilage thickness, loading pattern, immune response, and lifespan between small animals and humans may limit direct clinical extrapolation.

Therefore, positive findings from preclinical OA models should not be directly equated with clinical efficacy in patients. Future SVF‐gel studies should include multiple complementary OA models, including inflammatory, aging‐related, obesity/metabolic, and large‐animal models, with longer follow‐up periods and standardized structural, molecular, and functional endpoints. Emerging human‐relevant platforms, such as OA organoids and organoid‐based intelligent manufacturing systems, may provide additional tools to model the cartilage‐synovium‐subchondral bone microenvironment, evaluate disease heterogeneity, and improve translational prediction [72]. Moreover, recent evidence using DMM models suggests that donor‐related factors, such as donor age, may markedly influence the therapeutic efficacy of MSC‐derived extracellular vesicles [73]. These findings further highlight the need to consider donor variability, senescence, and disease‐stage differences when translating SVF‐gel‐based therapies from animal models to clinical OA treatment.

### 4.2. Clinical Studies

Current clinical evidence regarding the direct application of SVF‐gel in OA remains limited and is still at an early translational stage. Nevertheless, accumulating evidence from SVF‐based clinical studies provides important indirect support for the safety, feasibility, and regenerative potential of this therapeutic strategy. In OA, multiple prospective and retrospective clinical studies involving more than 3000 patients have evaluated the effects of intra‐articular SVF‐based therapy. Across these studies, the average follow‐up period was ~12 months. Therapeutic outcomes were commonly assessed using MRI‐based structural evaluation, the Western Ontario and McMaster Universities OA Index (WOMAC), and the visual analog scale (VAS) for pain. Overall, more than 75% of patients showed clinically meaningful improvement, including pain relief, improved joint function, and favorable structural changes. These findings suggest that SVF‐based therapy may provide symptomatic benefits and may potentially contribute to tissue repair in patients with OA [74].

However, most available clinical studies are heterogeneous in design, preparation protocol, patient selection, disease stage, injection dose, and follow‐up duration. Moreover, many studies evaluate SVF rather than SVF‐gel specifically. Therefore, although these data support the translational potential of SVF‐gel, they should not be interpreted as definitive evidence of SVF‐gel efficacy in OA. Well‐designed RCTs are needed to determine whether the matrix‐rich gel structure of SVF‐gel provides additional clinical benefits over conventional SVF, isolated MSCs, HA, PRP, or placebo.

Beyond OA, SVF‐based therapies have been investigated in several regenerative medicine settings, further supporting their broader biological activity and clinical feasibility. In a prospective clinical study involving 30 patients with androgenetic alopecia, patients received SVF therapy and were followed for 6 months. The hair shaft diameter increased by 51.35%, while hair density improved by 16.09% relative to baseline, with both changes reaching statistical significance. These findings suggest that SVF may enhance follicular regeneration and improve hair quality [75].

SVF‐based therapy has also been evaluated in scar management. A recent meta‐analysis including 20 studies and 493 patients, with follow‐up durations ranging from 3 to 24 months, showed significant improvements in both the Patient and Observer Scar Assessment Scale and the Vancouver Scar Scale. In addition, the scar surface area and thickness were significantly decreased compared with baseline measurements. These results indicate that SVF‐based approaches may improve the scar appearance, tissue remodeling, and structural quality [76].

In tendon‐related disorders, another meta‐analysis of eight studies with follow‐up periods ranging from 6 to 28 months reported that SVF injection significantly improved clinical outcomes in patients with rotator cuff disease and other tendinopathies. Significant improvements were observed in pain and functional scores, including the VAS, American Shoulder and Elbow Surgeons score, and numerical pain score. MRI evaluation also revealed improved tendon integrity and structural healing after the treatment. These findings suggest that SVF therapy may promote tendon regeneration and functional recovery, highlighting its potential as a regenerative strategy for musculoskeletal disorders [77].

For OA, the assessment of therapeutic efficacy should not be limited to biological or imaging‐based outcomes, such as inflammatory mediators, cartilage thickness, or structural repair. Clinically relevant and patient‐centered outcomes are equally important because the ultimate therapeutic goal of OA treatment is to relieve pain, restore joint function, improve mobility, and enhance quality of life. Therefore, future clinical studies of SVF‐gel should incorporate standardized functional and patient‐reported outcome measures, such as the VAS for pain, the WOMAC, the knee injury and OA outcome score (KOOS), and general quality‐of‐life instruments such as the Short Form‐36 (SF‐36) or EuroQol‐5 Dimension (EQ‐5D). These measures would allow a more comprehensive evaluation of whether biological or structural improvements translate into meaningful clinical benefits for patients.

Overall, the accumulating body of evidence suggests that SVF‐gel is a promising regenerative therapy with applications potentially extending beyond cartilage repair. Existing studies support the safety and feasibility of SVF‐based approaches, but direct clinical evidence for SVF‐gel in OA remains insufficient. Large‐scale, multicenter, RCTs with standardized SVF‐gel preparation protocols, defined dosing regimens, appropriate control groups, longer follow‐up, and comprehensive clinical and imaging outcomes are required to establish its long‐term safety and therapeutic efficacy.

## 5. Combined Application of SVF‐Gel With Other Therapeutic Strategies

Although SVF‐gel has shown great potential as a stand‐alone therapy for OA, its therapeutic effects may be further optimized when combined with adjunctive agents that provide complementary biomechanical, biochemical, and immunomodulatory support. The rationale for combination therapy should not be understood as a simple superposition of multiple regenerative products but rather as a strategy to simultaneously regulate the mechanical environment, synovial immune microenvironment, chondrocyte metabolism, ECM remodeling, and cartilage‐synovium crosstalk within the osteoarthritic joint. This concept is particularly relevant because synovial cells are closely involved in joint pain, inflammation, and cartilage destruction. In addition, recent evidence indicates that senescent synovial macrophages may exacerbate OA through enhanced M1 polarization, mitochondrial dysfunction, impaired efferocytosis, and increased senescence‐associated secretory phenotype secretion [78]. Therefore, combination strategies involving SVF‐gel should be evaluated not only in terms of cartilage repair but also in terms of their capacity to reshape the synovial inflammatory and immune microenvironment.

HA is widely used as an intra‐articular injection to protect cartilage by improving joint lubrication, restoring viscoelasticity, and reducing mechanical stress within the articular cavity. When combined with SVF‐gel, HA may complement the intrinsic biological matrix of SVF‐gel by improving rheological properties, enhancing lubrication, optimizing injectability, and promoting more favorable intra‐articular distribution and retention [74]. Mechanistically, HA may reduce friction‐related cartilage wear and attenuate mechanical stimulation of synovial cells and chondrocytes. In addition, HA can interact with cell‐surface receptors such as CD44 on synoviocytes, chondrocytes, and immune cells, thereby potentially influencing inflammatory signaling, matrix‐degrading enzyme expression, and synovial homeostasis. Since synovial cells and cartilage‐synovium crosstalk [79] are closely involved in OA progression, modulation of the synovial microenvironment may be an important mechanism underlying the benefit of HA. In this combination, SVF‐gel may provide cellular, ECM‐based, and paracrine support, whereas HA mainly contributes to mechanical protection, improved rheological behavior, enhanced intra‐articular distribution, and prolonged local retention. Thus, the potential additive effect of HA and SVF‐gel may arise from the integration of mechanical protection and biological repair. However, possible antagonistic effects should also be considered. Excessive HA concentration or viscosity may theoretically restrict cell migration, alter the diffusion of SVF‐gel‐derived paracrine factors, or affect the spatial distribution of the SVF‐gel within the joint cavity. Moreover, HA with different molecular weights may exert distinct biological effects on inflammation and cell behavior. Therefore, the optimal HA molecular weight, concentration, mixing ratio, and administration schedule should be clarified in future studies.

PRP is another promising adjunct because it contains a concentrated mixture of bioactive molecules, including PDGF, TGF‐β, VEGF, IGF‐1, FGF, EGF, and other platelet‐derived mediators. These factors may stimulate cell proliferation, migration, chondrogenic differentiation, ECM synthesis, and tissue repair. When combined with SVF‐gel, PRP may act as an early biochemical activator, whereas SVF‐gel may serve as a cellular and matrix‐based regenerative reservoir. The MSCs, EPCs, macrophages, ECM components, and soluble mediators in the SVF‐gel may respond to PRP‐derived signals, potentially enhancing paracrine activity, chondroprotective effects, and immunomodulatory responses. Moreover, the fibrin network formed after PRP activation may further support cell retention and localized factor release, thereby enhancing the residence time and biological activity of SVF‐gel in the joint cavity.

Nevertheless, the interaction between PRP and SVF‐gel may be context‐dependent and not necessarily synergistic in all settings. PRP preparations are highly heterogeneous, and differences in the platelet concentration, leukocyte content, activation method, and growth factor profile may substantially influence biological outcomes. Leukocyte‐rich PRP may aggravate inflammation in some contexts, whereas excessive or poorly controlled growth factor release may theoretically promote fibrosis, abnormal ECM deposition, or pathological angiogenesis. This is particularly important in OA, where synovial inflammation, senescent macrophages, and aberrant angiogenesis may contribute to pain sensitization and disease progression. Therefore, the combination of PRP with SVF‐gel should be optimized carefully rather than assumed to be universally beneficial. Current evidence suggests promising preclinical effects of adipose‐derived injectable products combined with PRP, but clinical evidence remains insufficient and inconsistent [80].

Overall, combination strategies involving SVF‐gel, HA, and PRP may offer therapeutic advantages by integrating lubrication, mechanical protection, immunomodulation, paracrine activation, and matrix‐based regenerative support. However, these effects are likely to depend on the disease stage, synovial inflammatory status, macrophage polarization and senescence status, SVF‐gel composition, HA molecular characteristics, PRP formulation, dose, timing, and injection schedule. Future studies should clarify the mechanistic interactions among these components and systematically evaluate both additive and antagonistic effects using standardized in vitro models, animal studies, and well‐designed clinical trials. In addition to biological and imaging markers, these studies should include patient‐centered outcomes such as pain relief, joint function, mobility, and quality of life.

## 6. Limitations and Challenges in Current Research

Although SVF‐gel shows promising prospects for the treatment of OA, the clinical and research use of SVF‐gel is limited due to the following issues and challenges, which should be addressed before its wider clinical application.

### 6.1. Preparation and Standardization of SVF‐Gel

The inherent heterogeneity of autologous adipose tissue, the cellular source of SVF‐gel, has been shown to be a significant factor impeding the standardization of SVF‐gel as a medical device. Factors such as the donor age, body mass index, as well as the anatomical origin of adipose tissue, significantly influence the cell profile, ECM composition, and pro‐inflammatory characteristics of the prepared gel. Each of these factors might have an impact on its therapeutic efficacy and on the safety profile, given that the risk of adverse events may vary according to the source of fat used.

The heterogeneity is additionally exacerbated by the preparation standards. This lack of standardization includes variations in collagenase concentration, digestion duration, tissue‐to‐enzyme ratio, centrifugation conditions, and the composition of the medium used to terminate enzymatic digestion. Different protocols for sample processing by laboratories and clinicians result in variation in yields and bioactivity as well as overall regenerative efficacy, thereby compromising the reproducibility and generalizability of the SVF‐gel for broader clinical applications.

Therefore, future efforts should be devoted to the introduction of reproducible, standardizable, and clinically scalable preparation procedures and well‐controlled quality control in order to guarantee that the different lots produced have uniform characteristics. Similarly, we also consider that scalable and clinical‐grade manufacturing approaches need to be devised and implemented to pave the way for the controlled, broad implementation of SVF‐gel in regenerative medicine.

### 6.2. Unclear Biological Mechanisms of SVF‐Gel

Although SVF‐gel shows great therapeutic potential, its precise mechanism of action remains incompletely defined. SVF‐gel is a complex mixture of stromal cells, immune cells, ECM components, and soluble mediators, and its biological effects may result from individual components, synergistic interactions, or both. At present, the relative contributions of MSCs, EPCs, macrophages, lymphocytes, ECM components, and secreted factors to cartilage repair, synovial immune regulation, and joint microenvironment remodeling remain poorly understood. Fundamental questions remain: are the therapeutic effects of SVF‐gel mainly driven by MSC‐mediated paracrine signaling, ECM‐mediated cell retention and mechanical support, immune‐cell‐mediated anti‐inflammatory effects, or the integrated interaction among these components? How does the ECM regulate the survival, localization, and secretory activity of the embedded cells? Which SVF‐gel‐derived factors act on chondrocytes, synoviocytes, macrophages, and other host cells within the osteoarthritic joint?

Importantly, direct comparative evidence remains limited. Few studies have systematically compared intact SVF‐gel with isolated MSCs, SVF cells without ECM support, ECM‐only or decellularized matrices, conditioned medium, extracellular vesicles, or other isolated components under the same experimental conditions. Such head‐to‐head comparisons are essential to determine whether SVF‐gel offers unique advantages beyond the simple combination of its parts. Future studies should combine comparative in vitro assays, animal models, single‐cell sequencing, spatial transcriptomics, proteomics, and loss‐of‐function or component‐depletion strategies to identify the key therapeutic components and their interactions. These studies would help clarify whether SVF‐gel should be optimized as an integrated multicellular matrix system or whether selected components can be engineered to achieve similar or superior therapeutic effects.

This multicellular and heterogeneous composition also presents a major barrier to the future application of precision engineering approaches such as gene editing because selective delivery of editing tools to specific target cell populations within SVF‐gel remains technically difficult. Without sufficient targeting specificity, unintended modification of nontarget cells, particularly immune cells, may lead to unpredictable biological effects and safety concerns.

The pathological OA development is a multifactorial process that consists of several stages: an inflammation predominant stage and a progression stage of cartilage deterioration. The therapeutic mechanisms of SVF‐gel may vary according to the disease stage. In the early stage, its anti‐inflammatory and immunomodulatory effects may be more prominent, whereas in the later stage, cartilage repair and regenerative effects may play a greater role. However, currently, there is limited systematic research exploring these stage‐specific therapeutic effects. Our in‐depth insight into how SVF‐gel performs at various stages of OA will allow us to optimize its clinical use and enhance its clinical effect. Besides, particular attention should be paid to defining the boundary between beneficial vascular remodeling and pathological angiogenesis in OA so that SVF‐gel‐based interventions can be optimized without aggravating synovitis, osteophyte formation, or pain‐related pathological changes.

### 6.3. Evaluation of Safety and Efficacy

Despite the safety and preliminary efficacy of SVF‐gel in OA treatment demonstrated in preclinical animal studies and early clinical investigations, several issues remain incompletely characterized. First, preclinical efficacy should be interpreted with caution because commonly used OA animal models, including chemically induced and surgically induced models, may not fully reproduce the chronic inflammatory, metabolic, mechanical, and aging‐related features of human OA. The primary clinical limitation is the absence of long‐term evidence, as most available studies have relatively short follow‐up periods, and data on long‐term effectiveness and safety remain limited [81]. Moreover, current evaluations are often focused on biological, histological, or imaging‐related outcomes, whereas clinically meaningful endpoints, including pain relief, joint function, physical activity, and quality of life, are less consistently assessed. This limits the ability to determine whether mechanistic or structural improvements can be translated into meaningful patient benefits. In addition to these efficacy‐related limitations, several safety‐related issues require further attention. Preparation‐related risks should be carefully considered. Some SVF‐based products are prepared using enzymatic digestion, whereas SVF‐gel is often obtained through mechanical processing and centrifugation. When enzymatic digestion is involved, residual enzymes, such as collagenase, may represent a potential source of local irritation, ECM disruption, or inflammatory reactions after intra‐articular injection [82]. Although washing procedures are generally used to minimize enzyme residues, the acceptable residual level and its potential effects on articular cartilage and synovial tissues remain insufficiently defined.

The safety of repeated intra‐articular administration is another important issue. Repeated injections may increase procedure‐related risks, including infection, post‐injection pain, effusion, synovitis, bleeding, and mechanical damage to intra‐articular structures [83]. Moreover, repeated exposure to adipose‐derived cellular and ECM components may theoretically modify the local immune microenvironment. However, current evidence is inadequate to determine whether repeated SVF‐gel injections carry additional risks compared with a single administration. Furthermore, the long‐term biocompatibility of SVF‐gel degradation products remains poorly understood. After injection, cellular components, ECM fragments, lipid residues, and soluble mediators may be degraded, remodeled, or cleared from the joint cavity. Their degradation products may theoretically contribute to persistent inflammation, abnormal remodeling, ectopic calcification/ossification, or other local adverse reactions, although direct evidence is still limited [84]. Therefore, future studies should include systematic safety assessments of residual preparation reagents, sterility, endotoxin levels, repeated administration, immunogenicity, ectopic tissue formation, and the metabolic fate and biotoxicity of degradation products.

Overall, large‐scale, long‐term, multicenter clinical studies with standardized preparation protocols, extended follow‐up, and comprehensive outcome assessment are needed to clarify the clinical safety and efficacy of SVF‐gel for OA treatment. Such studies should incorporate both objective biological or imaging markers and standardized patient‐centered outcomes to comprehensively determine the therapeutic value of the SVF‐gel.

### 6.4. Technical and Cost Barriers to Clinical Implementation

At present, the preparation of SVF‐gel requires a high technical qualification, strictly sterile environment, and special equipment with intricate operation. The technical requirements bring heavy economic burden on treatment, and only large hospitals can afford such measures. Generally speaking, these will also form an obstacle to large‐scale clinical application. In this regard, it is expected that automatic preparation platforms will be a research direction in the future. These systems could make production easier, reduce human‐related variance, and standardize procedures. Concomitantly, improvements in the preparation devices and procedures for reducing the cost will be key for widespread use of SVF‐gel in cost‐sensitive settings. Streamlining of preparation is also desirable, to simplify the preparation process and reduce technical barrier, making it accessible in primary care settings. Such technical and cost barriers will be key for advancing SVF‐gel as a more cost‐effective and convenient therapeutic for OA treatment and other regenerative applications.

## 7. Future Perspectives and Research Directions

### 7.1. Deepening Mechanistic Studies of SVF‐Gel

To further apply SVF‐gel in OA therapy, more insights regarding its therapeutic mechanisms are necessary. SVF‐gel is a complex biomaterial consisting of MSCs, ECM, and multiple growth factors, and the interactions among these components are highly complex. Thus, the interactions between its constituent elements should be investigated to optimize the therapeutic potential of SVF‐gel. Furthermore, as OA patients show a wide range in their severity of cartilage defects and disease stage, personalized therapeutic strategies using SVF‐gel should be employed for such patients. As an example, during early OA, one might be interested in stimulating its anti‐inflammatory and regulatory immune properties, whereas in the late stage, it may be cartilage repair and regeneration. In view of the effective treatment, it is reasonable for future studies to determine whether SVF‐gel functions more effectively in early‐stage or late‐stage OA, and tailored treatment strategies should be developed according to a specific stage.

### 7.2. Integration of SVF‐Gel With Multidisciplinary Technologies

The CRISPR/Cas9 technology also makes it possible to modify key chondrogenic signaling pathways in a targeted manner, including SOX9 and TGF‐β/Smad [85]. Targeting these pathways may allow gene editing to modulate the molecular activity of MSCs within the SVF‐gel and enhance their chondrogenic potential.

Gene‐editing technologies such as CRISPR/Cas9 may offer future opportunities to modulate key chondrogenic signaling pathways, including SOX9 and TGF‐β/Smad, and thereby potentially enhance the reparative activity of selected cell populations within the SVF‐gel. However, this strategy should be interpreted cautiously. SVF‐gel is a heterogeneous multicellular system containing MSCs, EPCs, macrophages, T cells, and ECM components, which makes precise cell‐specific delivery of gene‐editing tools particularly challenging [86]. If editing tools cannot be selectively delivered to the intended target cells, nonspecific editing may alter immune cell function or other critical biological activities, thereby raising safety concerns. Therefore, targeted delivery, editing specificity, and biosafety should be regarded as major obstacles that must be resolved before gene editing can be considered a practical strategy for SVF‐gel‐based therapy. Emerging precision‐editing approaches, including prime editing, may provide improved specificity in the future, but their applicability in complex multicellular biomaterials such as SVF‐gel remains to be fully established.

Concurrently, nanomaterials and 3D printing provide promising solutions to enhance the efficiency of SVF‐gel therapy. 3D printing of SVF‐gel could facilitate the construction of custom‐made biological scaffolds and generate an optimal microenvironment for the attachment, growth, and differentiation of cells [50, 87]. The addition of nanomaterials can also enhance the mechanical strength, biodegradability, and controlled release of growth factors for SVF‐gel. In all, these cutting‐edge technologies will lead the way to generate advanced, functionally enhanced SVF‐gel constructs for the future of personalized and intelligent regenerative therapies.

### 7.3. Standardization of Clinical Applications

Standardization of treatment guidelines and SVF‐gel preparation protocols should be set before SVF‐gel can be made more accessible as a clinical tool. Currently, different results due to different techniques of preparing and administering SVF‐gel could only be expected since no distinct guidelines of standard practices have been proposed or suggested to obtain consistent results. The patient inclusion criteria, dosage, administration intervals, and preparation methods could all benefit from being standardized. High‐quality clinical evidence from large‐scale, multicenter RCTs is needed to establish the safety and efficacy of SVF‐gel. The evidence obtained should later be used and transformed in the form of evidence‐based guidelines for the consistent and reliable application of SVF‐gel in OA treatment.

### 7.4. Personalized and Precision Treatment

The emergence of precision medicine provides a strong foundation to build personalized SVF‐gel‐based therapies for different patient profiles. It is beneficial to consider the disease stage, genetic profile, and clinical symptoms and include them when building treatments for individual patients. One aspect to take into account is the timing of the administration of the SVF‐gel since the inflammatory environment surrounding OA, throughout disease progression, will vary throughout its development. Early treatment with SVF‐gel during the acute inflammatory phase might be more effective in halting synovitis, whereas later administration could benefit more in limiting the chronic inflammation and enhancing healing. A thorough characterization of such time dependency effects is critical to tailor the benefit of clinical intervention.

New tools, such as artificial intelligence (AI) and big data analytics, provide opportunities to utilize individualized information (e.g., genomic and transcriptomic data) of patients in order to design individual treatments that are effective with the potential to lower the risk of side effects. Moreover, AI could be used to anticipate treatment reactions and to dynamically adapt interventions at the individual level to improve the precision and efficacy of SVF‐gel therapy. For instance, AI‐based imaging interpretation of synovial fluid composition, cartilage thickness, and inflammatory biomarkers could also be used to cluster patients into subgroups that are associated with having the same characteristics of their disease, and this could help tailor SVF‐gel, e.g., through altering levels in MSCs or growth factors, to fit that patient. Second, models could predict a patient’s treatment response given the biomarkers measured prior to treatment, enabling the clinician to update a treatment strategy dynamically to maximize therapeutic efficacy.

## 8. Conclusion

SVF‐gel is a stromal‐rich biomaterial containing ECM components and growth factors that has great potential for OA treatment because it can suppress inflammation, promote chondrocyte proliferation, and support tissue repair. Through these mechanisms, SVF‐gel may both ameliorate OA progression and potentially promote cartilage repair and potentially modify disease progression. There are still challenges that need to be addressed before SVF‐gel is widely used in clinical practice: consistent preparation and delivery methods should be established, its biological mechanisms should be better understood, and long‐term safety and efficacy data from longitudinal clinical trials are still needed. Importantly, future trials should evaluate not only biological and imaging markers but also patient‐centered outcomes, including pain, joint function, mobility, and quality of life, to determine the real‐world therapeutic value of SVF‐gel.

In future studies, the challenges of translating SVF‐gel into clinical OA management include better characterization of the roles of individual components in the response to SVF‐gel depending on the stages of OA development and integration with novel technologies such as gene editing technology, nanotechnology, and 3D printing. Among these emerging approaches, gene‐editing strategies should be viewed with particular caution, as the heterogeneous multicellular nature of SVF‐gel makes targeted delivery and cell‐specific editing difficult, and these limitations must be overcome before such strategies can be translated into practical clinical applications. Large clinical trials of multicenter design are required to drive the development of standard protocols in order to generate reliable results, which can ultimately be reproduced. The application of AI and big data to design individual patient‐specific treatment may provide further improvement of the outcome.

To successfully put SVF‐gel in mainstream clinical use will necessitate close cooperation amongst physicians, scientists, and engineers. Similarly, regulations and health policies need to be modified to accommodate novel regenerative therapies. To do so will be critical to successfully establishing SVF‐gel, alongside other promising regenerative treatment options, as a routine therapeutic option for OA and other degenerative diseases.

## Author Contributions

Runze Zhang, Wanli Gao, Xing Lei, and Xin Yin performed the literature search and data collection. Wei Zhang, Ling Zhang, Yuanhang Yang, and Yunqi Zhang contributed to the analysis and interpretation of the data. Runze Zhang and Junjun Yang drafted the initial manuscript.Junjun Yang, Lisheng Wu, and Maohua Chen conceptualized and supervised the study, critically reviewed the manuscript, and provided important intellectual input.

## Funding

This study was funded by the Chongqing Natural Science Foundation Postdoctoral Science Fund Project (Grant CSTB2023NSCQ‐BHX0156), the Linyi City Key Research and Development Program (Medical Category) (Grant 2024YX0065), the Hospital Emergency Project for Clinical Treatment and Scientific Research on COVID‐19 Infections (Grant 2023XGIIT09), the Shandong Province Postdoctoral Innovation Project (Grant SDCX‐ZG‐202400015), the Shandong Provincial Natural Science Foundation (Grant ZR2022MH071), and the Natural Science Foundation of Chongqing (Grant CSTB2024NSCQ‐MSX1142).

## Disclosure

All authors contributed to revising the manuscript, approved the final version, and agreed to be accountable for all aspects of the work.

## Conflicts of Interest

The authors declare no conflicts of interest.

## Data Availability

The data that support the findings of this study are available from the corresponding author upon reasonable request.
